# Genetic evaluation of BRCA1-A complex genes with triple-negative breast cancer susceptibility in Chinese women

**DOI:** 10.18632/oncotarget.7112

**Published:** 2016-02-01

**Authors:** Hong Ling, Shan Li, Yang Wu, Yi-Zi Zheng, Feng Qiao, Ling Yao, Zhi-Gang Cao, Fu-Gui Ye, Jiong Wu, Xin Hu, Bin Wang, Zhi-Ming Shao

**Affiliations:** ^1^ Department of Breast Surgery, Key Laboratory of Breast Cancer in Shanghai, Fudan University Shanghai Cancer Center, Fudan University, Shanghai, China; ^2^ Department of Oncology, Shanghai Medical College, Fudan University, Shanghai, China; ^3^ Key Laboratory of Medical Molecular Virology of Ministries of Education and Health, Institute of Medical Microbiology, Shanghai Medical College of Fudan University, Shanghai, China; ^4^ Department of Genetics, The University of Texas M.D. Anderson Cancer Center, Houston, Texas, United States

**Keywords:** BRCA1-A complex, NBA1, triple-negative breast cancer, polymorphism, cancer susceptibility

## Abstract

**Background:**

The tumor suppressor BRCA1 plays a pivotal role in maintaining genomic stability and tumor suppression. The BRCA1-A complex is required for recruitment of BRCA1 to DNA damage sites, DNA repair and cell cycle checkpoint control. Since germline mutations of *BRCA1* often lead to breast tumors that are triple-negative breast cancer (TNBC) type, we aimed to investigate whether genetic deficiency in genes of the BRCA1-A complex is associated with risk to TNBC development.

**Results:**

We found that rs7250266 in the promoter region of *NBA1* confers a decreased risk to TNBC development, but not to non-TNBC susceptibility. In addition, the haplotypes containing two polymorphisms rs7250266 and rs2278256 are associated with a lower chance of TNBC development specifically. Our studies also showed that the protective alleles of rs7250266 (C > G) and rs2278256 (T > C) down-regulate promoter activity of *NBA1* in mammary epithelial cells.

**Methods:**

We investigated associations between the BRCA1-A complex genes and TNBC developing risk in first case-control study of Chinese Han Women population including 414 patients with TNBC and 354 cancer-free controls. We detected 37 common variants in ABRAXAS, RAP80, BRE, BRCC36 and *NBA1/MERIT40* genes encoding the BRCA1-A complex and evaluated their genetic susceptibility to the risk of TNBC. An additional cohort with 652 other types of breast cancer (non-TNBC) cases and 890 controls was used to investigate the associations between TNBC-specific SNPs genotype and non-TNBCs susceptibility.

**Conclusions:**

Genetic variants in *NBA1* may be an important genetic determinant of TNBC susceptibility. Further investigation and validation of these SNPs in larger cohorts may facilitate in predication and prevention of TNBC and in counseling individuals for risk of TNBC development.

## INTRODUCTION

Breast cancer is a highly heterogeneous disease with distinct molecular and clinical phenotypes. Human breast tumors can be characterized into four major molecular subtypes: Luminal A, Luminal B, HER2 type, and Basal-like [1, 2]. Basal-like breast tumors, in general, have the worst prognosis, higher histological grade and poorer survival [1, 3]. A recent comprehensive analysis of The Cancer Genome Atlas (TCGA) program showed that around twenty percent of basal-like breast tumors have an inherited or somatic *BRCA1* or *BRCA2* nucleic acid variant [4]. Thus, the deleterious mutations in *BRCA1/BRCA2* are tightly associated with development of TNBC. It is suggested that genetic variant of multiple low-risk polymorphisms of genes encoding *BRCA1/BRCA2* interacting proteins may also be associated with risk of TNBC [5–10].

During the past decades, pathogenic mutations of *BRCA1* have been widely investigated in etiologic studies in breast and ovarian cancer. BRCA1 suppresses malignant transformation at least partially through regulating the DNA damage response and maintaining genome stability [11, 12]. The BRCA1-A complex directly interacts with the BRCT domains of BRCA1 and mediates BRCA1 protein accumulation to DNA damage sites [12–16]. The BRCA1-A complex contains at least five protein components ABRAXAS, RAP80, BRE, BRCC36 and NBA1/MERIT40 [12, 17–19].

ABRAXAS appears to serve as a central adaptor protein in the BRCA1-A complex bridging the interactions of each member of the complex with BRCA1 [13, 14, 18]. RAP80 contains a tandem SUMO interacting (SIM)-ubqiuitin interacting (UIM)-UIM motif which displays binding specificities toward both Lys-63 linkage ubiquitin conjugates and SUMO2 conjugates [20–23]. In the BRCA1-A complex, BRCC36 is a de-ubiquitinating enzyme (DUB) which has a de-ubiquitinating activity specifically toward K63-polyUb linkages [24]. NBA1/MERIT40 and BRE is also identified as a BRCA1 associated protein which contains a VWA domain and two UEV domains, respectively [14, 19, 25]. Our recent work uncovered that NBA1interacts with BRE is critical for maintaining the integrity of the BRCA1-A complex and cellular resistance to ionizing radiation [18].

Our previous studies showed that germline *BRCA1* and *BRCA2* mutations are associated with early onset breast cancer and familial breast cancer in Chinese women population [26–30]. Several studies indicate that single-nucleotide polymorphisms (SNPs) in locus 19p13.1 including rs8170 and rs3745185 in *NBA1* gene are associated with risk of breast cancer [6, 31]. Two recent genome-wide association studies (GWAS) have identified the locus of 19p13.1 is associated with risk of developing hormone receptor–negative breast cancer and ovarian cancer [5, 32]. It was worth noting that most previous studies of common variants in BRCA1-A complex genes were investigated in European ancestry populations; in contrast, the associations of polymorphisms in these genes and the risks of TNBC development have not been thoroughly investigated in Chinese women population.

In step-one analysis, we performed a case-control study to examine 37 common genetic variants of the BRCA1-A complex genes in patients with TNBC in Chinese women population. Our result revealed that rs7250266 in NBA1 was associated with decreased risk of developing triple-negative breast cancer. Haplotypes containing two polymorphisms rs2278256 and rs7250266 within promoter region of *NBA1* were also correlated to a lower chance of triple-negative breast cancer development. Further *in silico* and biochemical analysis demonstrated that these protective alleles of rs7250266 (C > G) and rs2278256 (T > C) could markedly down-regulate the promoter activity of NBA1in mammary epithelial cells. In step-two analysis, we recruited 652 breast cancer patients with other types of breast cancer and 890 normal women as controls in the second cohort. We tested the phenotype of rs2278256 and rs7250266 in this cohort, and found no difference between non-TNBCs and ordinary people. We herein demonstrated these two SNPs (rs2278256 and rs7250266) were tightly associated with an decreased risk of developing TNBC, but not with non-TNBCs susceptibility.

## RESULTS

### Screen for variants of the BRCA1-A complex genes in triple-negative breast cancer patients and controls

We carried out a systematic analysis of 37 genetic variants in genes from the BRCA1 associated A complex including *ABRAXAS*, *BRCC36, RAP80 BRE* and *NBA1*, in a Chinese Han Women cohort, including 414 triple-negative breast cancer (TNBC) cases and 354 controls. The criteria for selected polymorphisms are described in “Materials and Methods” section. As shown in Supplementary Table S2, the patients' clinical characteristics in this study revealed that the TNBC cases were significantly more likely to be at menarche at younger age than the controls. We assessed their associations with TNBC using genotype data from SEQUENOM MassARRAY platform (Table 1). We observed no significant deviation from Hardy–Weinberg equilibrium of each polymorphism either in controls or in cases with a cut-off value of 0.05. All polymorphisms were genotyped successfully with genotyping call rate ranging from 95 to 100%.

**Table 1:** Allele frequencies of each SNPs in step-one cohort (414 TNBCs and 354 controls)

							MAF		
rs number	Gene	Chromosone	Position	Protein Change	Minor Allele	Reference Allele	Case(*n* = 828)	Control(*n* = 708)	*P*^a^ value
rs2278256	NBA1	19p13.11	5′ near gene		C	T	0.32	0.36	0.06
rs3745185	NBA1	19p13.11	Intron		A	G	0.13	0.11	0.21
rs10406920	NBA1	19p13.11	Intron		T	C	< 0.01	0	N/A
rs8170	NBA1	19p13.11	Exon	synonymous	T	C	0	0	N/A
***rs7250266***	***NBA1***	***19p13.11***	***5′ near gene***		***G***	***C***	***0.14***	***0.19***	***< 0.01***
rs144376330	NBA1	19p13.11	Exon	synonymous	T	C	0.01	0.01	0.88
rs10403581	NBA1	19p13.11	Intron		C	A	0.27	0.28	0.49
rs895745	Brcc36	Xq28	Intron		A	G	0.19	0.22	0.28
rs4898413	Brcc36	Xq28	Intron		T	A	0.19	0.22	0.29
rs5945286	Brcc36	Xq28	Intron		T	C	0.06	0.06	0.96
rs5945300	Brcc36	Xq28	Intron		G	A	0.18	0.21	0.17
rs12422	Rap80	5q35.2	3′UTR		G	T	0	0	N/A
rs3733876	Rap80	5q35.2	Exon	Missense	A	G	0.17	0.15	0.28
rs11739147	Rap80	5q35.2	Intron		C	T	0.36	0.35	0.82
rs365132	Rap80	5q35.2	Exon	synonymous	G	T	0.47	0.49	0.82
rs13360277	Rap80	5q35.2	Exon	Missense	G	A	0.02	< 0.01	0.30
rs353465	Rap80	5q35.2	Intron		C	T	0.36	0.35	0.80
rs17078658	Rap80	5q35.2	Intron		G	T	0.11	0.1	0.45
rs17078630	Rap80	5q35.2	Intron		T	C	0	0	N/A
rs13167812	Rap80	5q35.2	Exon	Missense	A	G	0	0	N/A
rs6547829	BRE	2p23.2	Intron		T	C	0.17	0.16	0.72
rs12464240	BRE	2p23.2	Intron		T	C	0.44	0.45	0.94
rs17709034	BRE	2p23.2	Intron		T	C	0	0	N/A
rs6721349	BRE	2p23.2	Intron		T	C	< 0.01	< 0.01	0.94
rs58720304	BRE	2p23.2	3′UTR		C	T	0.38	0.36	0.58
rs12478271	BRE	2p23.2	Intron		T	C	< 0.01	< 0.01	0.58
rs10173507	BRE	2p23.2	Intron		C	T	0.3	0.3	0.96
rs6737313	BRE	2p23.2	Intron		G	A	0.44	0.45	0.74
rs6710214	BRE	2p23.2	Intron		G	A	0.27	0.29	0.54
rs10209126	BRE	2p23.2	Intron		T	C	0.36	0.34	0.62
rs11891642	BRE	2p23.2	Intron		T	C	< 0.01	< 0.01	0.86
rs10189899	BRE	2p23.2	Intron		A	G	0.35	0.33	0.25
rs77519137	Abraxas	4q21.23	3′UTR		G	A	0	< 0.01	N/A
rs13125836	Abraxas	4q21.23	Exon	Missense	T	C	0	0	N/A
rs72931487	Abraxas	4q21.23	3′ near gene		G	A	0	0	N/A
rs12642536	Abraxas	4q21.23	Exon	Missense	C	T	0.32	0.33	0.45
rs17352824	Abraxas	4q21.23	Intron		G	A	0.32	0.34	0.35

Abbreviations: MAF, minor allele frequency; N/A, not applicable; UTR, untranslated region.

NOTE:

a Unadjusted *P*-value of two-sided χ2 test.

Bold values denote *P* ≤ 0.05.

### Identification of rs7250266 in *NBA1* as a SNP associated with decreased risk of TNBC in chinese han women population

Among the 37 polymorphisms we identified in the BRCA1-A complex genes, we found one polymorphism rs7250266, which is located at −776 nt at the 5′ promoter region of the *NBA1* gene, showed a statistically significant association with TNBC. The allelic frequency of the G-allele of rs7250266 was 0.19 in controls compared with 0.14 in patients with significant difference (*P* = 0.006, Table 1). As shown in Table 2, a comparison of genotype frequency between TNBC cases and controls showed that genotypes GC or GG of rs7250266 was associated with a significant decreased risk of TNBC in a co-dominant model (GC genotype, odds ratio (OR) = 0.70, 95% CI 0.51–0.97; GG genotype, OR = 0.48, 95% CI 0.21–1.07, *P* = 0.03). Under a dominant model, it's shown that women with genotypes GC or GG of rs7250266 conferred approximately 33% decreased risk to the development of TNBC (OR = 0.67, 95% CI 0.49– 0.92, *P* = 0.01).

**Table 2:** Associations between each SNPs genotypes and TNBC risk (414 TNBCs and 354 controls)

				Codominant		Dominant		Recessive	
SNP	Genotype	Case (%)	Control (%)	OR (95% CI)	*P*	OR (95% CI)	*P*	OR (95% CI)	*P*
rs2278256	TT	47.8	41.1	reference	0.16	reference	0.06	reference	0.29
	TC	41.3	45.4	0.78 (0.58–1.06)		0.76 (0.57–1.02)		0.79 (0.51–1.22)	
	CC	10.9	13.4	0.70 (0.44–1.11)					
rs3745185	GG	75.4	79.6	reference	0.36	reference	0.17	reference	1.00
	AG	22.9	18.7	1.29 (0.91–1.84)		1.27 (0.90–1.79)		1.00 (0.33–3.01)	
	AA	1.7	1.7	1.06 (0.35–3.18)					
***rs7250266***	***CC***	***74.3***	***67.1***	***reference***	***0.03***	***reference***	***0.01***	***reference***	***0.11***
	***GC***	***23.3***	***29.5***	***0.70 (0.51–0.97)***		***0.67 (0.49–0.92)***		***0.52 (0.23–1.17)***	
	***GG***	***2.4***	***4.5***	***0.48 (0.21–1.07)***					
rs144376330	CC	97.6	97.7	reference	0.88	reference	N/A	reference	N/A
	CT	2.4	2.3	1.08 (0.42–2.76)		N/A		N/A	
	TT	0	0	N/A					
rs10403581	AA	55.1	53.7	reference	0.70	reference	0.70	reference	0.40
	CA	36.6	36.3	0.98 (0.73–1.34)		0.95 (0.71–1.26)		0.81 (0.49–1.33)	
	CC	8.2	10	0.80 (0.48–1.34)					
rs895745	GG	64.5	61.6	reference	0.44	reference	0.42	reference	0.24
	AG	32.4	33.5	0.92 (0.68–1.25)		0.89 (0.66–1.19)		0.64 (0.31–1.34)	
	AA	3.2	4.8	0.63 (0.30–1.32)					
rs4898413	AA	64.5	61.8	reference	0.45	reference	0.44	reference	0.24
	AT	32.4	33.4	0.93 (0.68–1.26)		0.89 (0.66–1.19)		0.65 (0.31–1.35)	
	TT	3.2	4.8	0.63 (0.30–1.32)					
rs5945286	CC	87.3	88.3	reference	0.07	reference	0.68	reference	N/A
	TC	12.7	10.8	1.18 (0.76–1.84)		1.10 (0.71–1.69)		N/A	
	TT	0	0.8	N/A					
rs5945300	AA	66	62.9	reference	0.15	reference	0.36	reference	0.06
	GA	32	32.9	0.93 (0.68–1.26)		0.87 (0.65–1.17)		0.44 (0.19–1.06)	
	GG	1.9	4.3	0.43 (0.18–1.04)					
rs3733876	GG	69.4	73.2	reference	0.52	reference	0.25	reference	0.79
	AG	27.4	24	1.20 (0.87–1.67)		1.20 (0.88–1.65)		1.12 (0.49–2.59)	
	AA	3.2	2.8	1.18 (0.51–2.73)					
rs11739147	TT	38.8	41.6	reference	0.43	reference	0.43	reference	0.45
	TC	51	46.5	1.18 (0.87–1.59)		1.12 (0.84–1.50)		0.84 (0.53–1.32)	
	CC	10.2	11.9	0.92 (0.57–1.49)					
rs365132	TT	26.2	26.4	reference	0.19	reference	0.95	reference	0.09
	GT	53.6	48.3	1.12 (0.80–1.58)		1.01 (0.73–1.40)		0.75 (0.53–1.05)	
	GG	20.1	25.3	0.80 (0.53–1.21)					
rs353465	TT	38.5	41.5	reference	0.42	reference	0.41	reference	0.46
	TC	51.2	46.6	1.18 (0.87–1.60)		1.13 (0.85–1.51)		0.84 (0.54–1.33)	
	CC	10.2	11.9	0.92 (0.57–1.50)					
rs17078658	TT	78.5	81	reference	0.66	reference	0.39	reference	0.85
	GT	20.7	18.1	1.18 (0.82–1.69)		1.17 (0.82–1.66)		0.86 (0.17–4.29)	
	GG	0.7	0.8	0.89 (0.18–4.44)					
rs6547829	CC	68	71.3	reference	0.07	reference	0.33	reference	0.06
	CT	30.8	25.6	1.26 (0.92–1.73)		1.17 (0.86–1.59)		0.38 (0.13–1.10)	
	TT	1.2	3.1	0.41 (0.14–1.18)					
rs12464240	CC	30.8	30.5	reference	1.00	reference	0.94	reference	0.98
	CT	49.6	49.7	0.99 (0.71–1.37)		0.99 (0.73–1.35)		1.00 (0.75–1.32)	
	TT	19.6	19.8	0.98 (0.65–1.48)					
rs58720304	TT	38.6	38.6	reference	0.48	reference	1.00	reference	0.25
	CT	47.8	50.6	0.94 (0.70–1.28)		1.00 (0.75–1.34)		1.29 (0.83–2.00)	
	CC	13.6	10.9	1.25 (0.78–2.00)					
rs10173507	TT	49	47.4	reference	0.73	reference	0.66	reference	0.61
	CT	42	44.6	0.91 (0.68-1.23)		0.94 (0.71–1.25)		1.14 (0.68–1.91)	
	CC	9	8	1.09 (0.64–1.86)					
rs6737313	AA	31.1	30.5	reference	0.93	reference	0.87	reference	0.70
	AG	50.1	49.6	0.99 (0.72–1.38)		0.97 (0.71–1.33)		0.93 (0.65–1.34)	
	GG	18.8	19.9	0.93 (0.61–1.40)					
rs6710214	AA	53.3	49.6	reference	0.44	reference	0.31	reference	0.66
	AG	38.4	43	0.83 (0.62–1.12)		0.86 (0.65–1.15)		1.13 (0.66–1.92)	
	GG	8.3	7.4	1.04 (0.60–1.80)					
rs10209126	CC	42.5	40.7	reference	0.12	reference	0.63	reference	0.08
	TC	43.7	49.6	0.85 (0.62–1.15)		0.93 (0.70–1.24)		1.50 (0.95–2.35)	
	TT	13.8	9.7	1.37 (0.85–2.21)					
rs10189899	GG	41	43.8	reference	0.46	reference	0.45	reference	0.25
	GA	47.3	47.2	1.07 (0.79–1.45)		1.12 (0.84–1.49)		1.32 (0.82–2.11)	
	AA	11.7	9.1	1.37 (0.83–2.25)					
rs12642536	TT	46.7	44.7	reference	0.72	reference	0.58	reference	0.46
	CT	43.5	43.9	0.95 (0.70–1.28)		0.92 (0.69–1.23)		0.84 (0.53–1.33)	
	CC	9.7	11.4	0.82 (0.50–1.33)					
rs17352824	AA	46.5	43.9	reference	0.61	reference	0.47	reference	0.39
	AG	43.8	44.4	0.93 (0.69–1.26)		0.90 (0.68–1.20)		0.82 (0.51–1.29)	
	GG	9.7	11.7	0.79 (0.48–1.28)					

Abbreviations: N/A, not applicable.

NOTE: ^a^ Unadjusted odds ratio (OR) and 95% confidential interval (CI) calculated by logistic regression. Bold values denote *P* ≤ 0.05.

The remaining polymorphisms identified in the BRCA1-A complex genes have no significant difference of MAFs between patients and controls. We found that two previously reported polymorphisms of the *NBA1* gene rs8170 and rs3745185 also exist in the cohort we studied. Although these two polymorphisms are associated with triple-negative breast cancers of *BRCA1* mutation carriers in previous GWAS studies of the European ancestry population [6, 31, 32], our results showed that there is no association between these two SNPs and risk of TNBCs in the Chinese Han women population.

### Characterization of SNPs identified in the BRCA1-A complex genes using linkage disequilibrium (LD) and haplotype analysis

**Figure 1:**
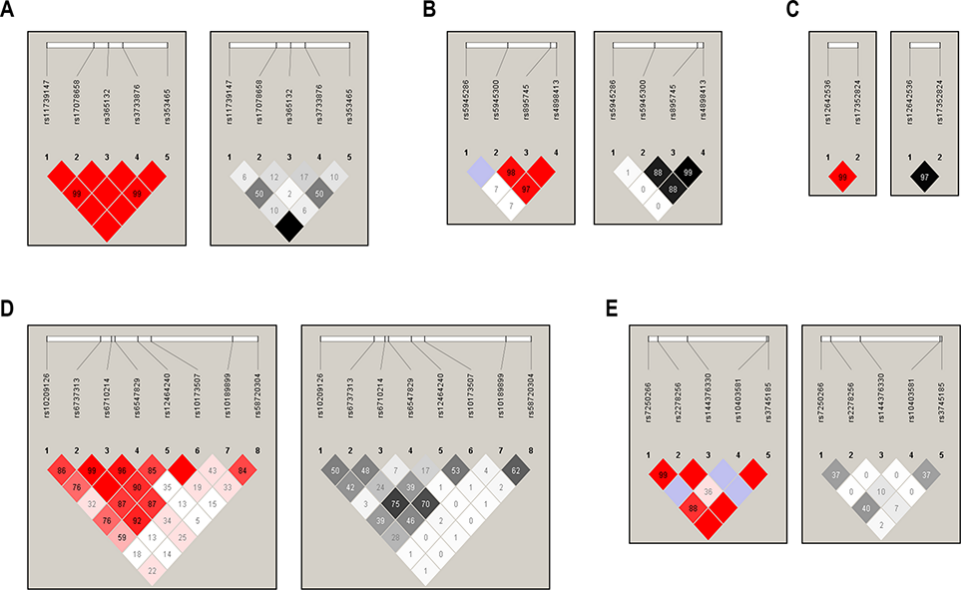
Linkage disequilibrium (LD) analysis of the BRCA1-A complex genes SNPs The top horizontal bar indicates the genetic region spanning the tested SNPs. The left triangle shows the LD calculated using the D' measure; the right triangle shows the LD calculated using the r2 measure. (**A**), LD-plot of RAP80; (**B**), LD-plot of BRCC36; (**C**), LD-plot of ABRAXAS; (**D**), LD-plot of BRE; (**E**), LD-plot of NBA1.) The value within each diamond represents the pairwise correlation between polymorphisms defined by the upper left and the upper right sides of the diamond. The red-to-white or black-to-white gradient reflects higher to lower LD values.

We then further characterized the 37 polymorphisms using linkage disequilibrium (LD) analysis of each BRCA1-A complex gene (Figure 1). Thirteen SNPs with MAF less than 0.01 in this study were excluded for LD characterization and haplotype analysis. We noticed that two *NBA1* variants rs2278256 and rs7250266 have a high linkage disequilibrium (LD) with a D' of 0.99 and r^2^ of 0.37 in the LD-plot. Additionally, some tested genetic variants in genes of BRCA1-A complex are in high LD, respectively (rs11739147, rs365132 and rs353465 in RAP80; rs5945300 and rs895745 in BRCC36; rs12642536 and rs17352824 in ABRAXAS; rs6737313 and 6710214 in BRE).

We also conducted haplotype analysis of SNPs in the BRCA1-A complex genes for the risk of developing TNBC. As shown in Table 3, we observed that haplotypes H3 and H5 in the *NBA1* gene were significantly associated with a decrease in risk of TNBC (H3, OR = 0.75, 95% Cl: 0.56–0.99, *P* = 0.04; H5, OR = 0.34, 95% Cl: 0.14–0.85, *P* = 0.02), respectively. Notably, these haplotypes contain two polymorphisms rs7250266 (c.-620 C > G) and rs2278256 (c.-73 T > C) within the 5′-promotor region of *NBA1*.

**Table 3:** Haplotype analysis of the investigated genes of the BRCA1-A complex in step-one cohort (414 TNBCs and 354 controls)

Locus	SNPs								Frequency	OR	95%CI	*P*
NBA1	rs7250266	rs2278256	rs144376330	rs10403581	rs3745185							
H1	C	T	C	A	G				0.528	1.00		
H2	C	C	C	A	G				0.172	0.99	0.75 – 1.32	0.97
***H3***	***G***	***C***	***C***	***C***	***G***				***0.149***	***0.75***	***0.56 – 0.99***	***0.04***
H4	C	T	C	C	A				0.122	1.14	0.83 – 1.57	0.41
***H5***	***G***	***C***	***C***	***A***	***G***				***0.015***	***0.34***	***0.14 – 0.85***	***0.02***
H6	C	T	T	A	G				0.012	1.00	0.38 – 2.58	0.99
H7	G	T	C	C	G				rare			
H8	C	C	C	C	G				rare			
H9	C	C	C	C	A				rare			
BRCC36	rs895745	rs4898413	rs5945286	rs5945300								
H1	G	A	C	A					0.744	1.00		
H2	A	T	C	G					0.188	0.87	0.67 – 1.13	0.28
H3	G	A	T	A					0.049	1.09	0.68 – 1.75	0.72
H4	A	T	T	A					0.014	0.77	0.32 – 1.84	0.55
H5	A	T	C	A					rare			
H6	G	A	C	G					rare			
H7	G	A	T	G					rare			
H8	A	A	C	G					rare			
H9	A	T	T	G					rare			
Rap80	rs3733876	rs11739147	rs365132	rs353465	rs17078658							
H1	G	T	G	T	T				0.374	1.27	0.88 – 1.81	0.20
H2	G	C	T	C	T				0.352			
H3	A	T	T	T	T				0.15			
H4	C	A	C	C	A				0.158			
H5	G	T	G	T	G				0.106			
H6	G	T	T	T	T				rare			
H7	G	C	G	C	T				rare			
H8	A	C	T	C	T				rare			
H9	A	T	G	T	T				rare			
BRE	rs6547829	rs12464240	rs58720304	rs10173507	rs6737313	rs6710214	rs10209126	rs10189899				
H1	C	C	T	T	A	A	C	G	0.281	1.00		
H2	C	C	C	T	A	A	C	A	0.182	1.30	0.91 – 1.87	0.15
H3	C	T	T	C	G	G	T	G	0.159	1.17	0.81 – 1.69	0.39
H4	T	T	T	T	G	A	T	G	0.042	1.12	0.59 – 2.13	0.73
H5	C	T	C	C	G	G	T	A	0.037	0.87	0.42 – 1.80	0.71
H6	C	T	T	C	G	G	C	G	0.031	1.00	0.51 – 1.98	1.00
H7	C	C	C	T	A	A	C	G	0.023	0.79	0.34 – 1.80	0.57
H8	T	T	C	T	G	A	C	A	0.023	0.90	0.40 – 2.02	0.79
H9	T	T	C	T	G	A	T	A	0.021	1.36	0.55 – 3.40	0.50
H10	T	T	T	C	G	A	C	G	0.021	0.54	0.21 – 1.40	0.21
H11	C	C	T	T	A	A	T	G	0.018	2.23	0.80 – 6.24	0.13
H12	C	T	C	C	G	G	T	G	0.016	1.38	0.51 – 3.69	0.52
H13	C	C	T	T	A	A	C	A	0.014	2.19	0.62 – 7.72	0.22
H14	T	T	T	T	G	A	C	G	0.013	1.47	0.43 – 5.03	0.54
H15	T	T	T	T	G	A	T	A	rare			
H16	T	C	C	T	G	A	T	A	rare			
H17	C	C	T	T	G	G	T	G	rare			
H18	C	T	T	T	A	A	C	G	rare			
H19	T	T	C	T	G	A	C	G	rare			
H20	C	T	T	C	A	A	C	G	rare			
H21	C	T	C	T	G	G	T	A	rare			
H22	C	C	C	T	A	A	T	A	rare			
H23	C	T	C	T	A	A	C	G	rare			
H24	C	T	C	C	G	G	C	A	rare			
H25	C	T	C	C	G	G	C	G	rare			
H26	T	T	T	C	G	A	T	G	rare			
H27	T	T	C	C	G	A	C	A	rare			
H28	C	C	C	T	G	G	C	A	rare			
H29	C	T	T	C	G	G	T	A	rare			
H30	C	T	C	T	A	A	C	A	rare			
H31	C	T	C	C	A	A	C	A	rare			
H32	C	C	T	T	G	A	C	G	rare			
H33	T	T	C	T	G	G	T	A	rare			
Abraxas	rs12642536	rs17352824										
H1	T	A							0.672	1.00		
H2	C	G							0.323	0.92	0.74 – 1.14	0.42
H3	T	G							rare			
H4	C	A							rare			

NOTE: Bold values denote *P* ≤ 0.05.

In the haplotypes, bold indicates the SNP variants that represent the likely variants associated with the reported effects.

### rs7250266 and rs2278256 showed no association with risk of non-TNBC breast cancer in chinese han women population

In step-two analysis, we further investigate the association between rs7250266 and rs2278256 and non-TNBC breast cancer susceptibility via case-control cohort comprising 652 non-TNBC cases and 890 normal controls. The patients' clinical characteristics are listed in Supplementary Table S3. In this study, the allelic frequency of the G-allele of rs7250266 was 0.19 in controls compared with 0.18 in patients (*P* = 0.85). As shown in Supplementary Table S4, comparison of genotype frequency between non-TNBCs and controls showed that genotypes GC or GG of rs7250266 was not associated with risk of cancer in either co-dominant model (GC genotype, odds ratio (OR) = 0.94, 95% CI 0.75–1.18; GG genotype, OR = 1.09, 95% CI 0.64–1.86, *P* = 0.81) or dominant model (GC + GG genotype, OR = 0.96, 95% CI 0.77–1.19, *P* = 0.71). Also shown in Supplementary Table S4, rs2278256 showed no association with risk of non-TNBC breast cancer in Chinese Women population.

### Effects of polymorphisms rs7250266 and rs2278256 on *NBA1* promoter activity in mammary epithelial cells

To explore the pathogenicities of potential functional SNPs involved in BRCA1-A complex, we performed *in silico* functional predictions for SNPs located in 5′ promoter regions, in exons and in 3′ UTR regions (Supplementary Table S5). As rs7250266 and rs2278256 in *NBA1* exert their tight associations with TNBC susceptibility, *RegulomeDB*, *TFSEARCH* and *SNPinfo* were utilized to predict the function of these two polymorphisms in the *NBA1* promoter region. These programs all provided a similar prediction that allelic changes of rs7250266 and rs2278256 in the promoter region of NBA1 are likely to affect the binding ability of transcription factors and lead to expression changes of the gene.

**Figure 2:**
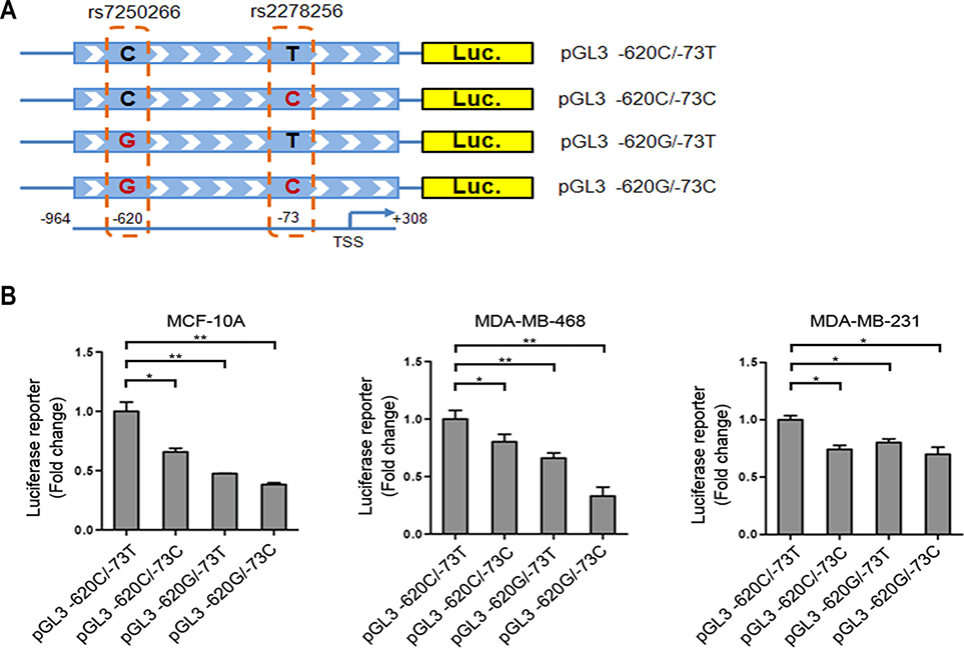
rs7250266 and rs2278256 reduce *NBA*1 promoter activity (**A**) Illustration of luciferase reporter constructs containing rs7250266 or rs2278256. DNA fragment of *NBA1* promoter region (−964 to +308 bp, the transcriptional start site is determined as +1) containing rs7250266 (C > G), rs2278256 (T > C) or both was cloned into pGL3 luciferase vector respectively. (**B**) Luciferase reporter assay of cells expressing constructs containing different alleles of rs7250266 and rs2278256. MCF-10A, MDA-MB-468 or MDA-MB-231 cells were transiently transfected with luciferase reporter constructs as illustrated and the internal pRL-CMV control plasmid. Luciferase activity from cells carrying constructs containing the C-allele of rs7250266 and T-allele of rs2278256 (pGL3 −620C/−73T) were set to “1” and used for control for comparison. Fold change was calculated by comparing luciferase activity from cells expressing other alleles of rs7250266 and rs2278256 to cells expressing pGL3 −620C/−73T. Data represent mean values, with error bars indicating S.E.M. (standard error of the mean). Statistical data were analyzed by the *t* test (*P < 0.05; **P < 0.01). Experiments have been repeated three times for each cell line and similar results were obtained.

To further examine whether these two SNPs in *NBA1* promoter region affect the promoter activity, we generated four promoter-reporter constructs containing rs7250266 (−620 C allele or G allele) or rs2278256 (−73 T allele or C allele) respectively (Figure 2A). Luciferase activity reporter assay showed that transcriptional activity of constructs carrying the G-allele of rs7250266, the C-allele of rs2278256 or both alleles displayed reduced promoter activity in several mammary epithelial cell lines (Figure 2B), suggesting that rs7250266 and rs2278256 determined the promoter activity of *NBA1* in mammary cells. This finding implicates that the protective alleles of rs7250266 (C > G) and rs2278256 (T > C) decrease NBA1 levels for a lower risk of developing TNBC.

## DISCUSSION

The BRCA1 BRCT domains are essential for BRCA1's tumor suppressor function [12, 17, 33]. At least three different BRCA1 BRCT domains associated complexes are identified, named as the BRCA1 A, B and C complexes, corresponding to the unique adaptor proteins that directly interact with the BRCT domains, ABRAXAS, BACH1 and CtIP (Figure S1) [12]. The BRCA1-A complex is known as a key mediator for recruitment of BRCA1 to DNA damage site and plays an important role in cell cycle checkpoint control and DNA damage repair. Thus it is likely that variants in the BRCA1-A complex genes impair the functions of BRCA1 and contribute to breast cancer susceptibility. Most of BRCA1-deficient breast cancer are triple-negative and basal-like. Considering that deficiency in the BRCA1-A complex could impair the biological functions of BRCA1, we chose a cohort of triple-negative breast cancer patients instead of a cohort of BRCA1 mutation carriers in this case-control study.

Several previous studies have carried out analysis of SNPs and screens for mutation in genes encoding BRCA1 interacting proteins including the BRCA1-A complex proteins (Supplementary Table S6). In the BRCA1-A complex, ABRAXAS is a central organizing adaptor protein that mediates the interaction of the BRCA1-A complex with BRCA1 [13, 16, 34, 35]. Solyom et al. reported a missense variant in *ABRAXAS*, c.1082G > A, is associated with an increased risk of breast cancer in familial breast/ovarian cancer women population [35–38]. RAP80 binds to both Lys63-linkage ubiquitin conjugates and SUMO conjugates and is required for the recruitment of the BRCA1-A complex to DNA damage sites [13, 20–23]. A missense variant (c.1304C > T) at the coding region of RAP80 was identified associated with increased risk to breast cancer [39]. BRCC36 is a MPN+/JAMM domain containing deubiquitinating enzyme with a catalytic activity specifically for K63-polyUb conjugates [24]. No significant SNP has yet been found in *BRCC36* gene so far [7]. BRE, also known as BRCC45, directly interacts with NBA1 in the BRCA1-A complex and the BRE-NBA1 interaction is essential for maintaining the integrity of BRCA1-A complex [18]. One SNP (rs11891642) in the intron region of *BRE* was reported associated with an increased risk of breast cancer in a *BRCA1* mutation carrier cohort [7]. NBA1/MERIT40 is identified as a novel BRCA1 associated protein which contains a VWA domain and a PxxR motif that directly interacts with BRE. Our study and several previous studies indicate that nucleic acid variants of *NBA1* are associated with risk of hormone negative breast cancer and advanced ovarian cancer in different ethnics [5, 7, 9, 31, 32]. The previous genome wide association studies (GWAS) showed that two SNPs (rs8170 and rs3745185) in *NBA1* are associated with a higher risk of TNBC in Caucasian population [7, 31, 32]. However, we found the MAFs of rs8170 and rs10406920 are very low in Chinese women population. Therefore, rs8170 and rs10406920 confer high risks of breast and ovarian cancer in Caucasian population, but not in Chinese population.

In this study, we identified that SNP rs7250266 in the promoter region of *NBA1* is associated with reduced risk to TNBCs. Another SNP rs2278256 in the promoter region of *NBA1*, despite its association with reduced risk of TNBC is borderline (*P* = 0.06), has a high linkage disequilibrium to rs7250266 using the LD construction method of Gabriel et al. (37). Furthermore, our analysis showed that haplotypes containing both protective alleles of rs7250266 (C > G) and rs2278256 (T > C), had a lower risk of developing TNBC. Considering that rs2278256 and rs7250266 locate in a high linkage disequilibrium with D'> 0.99, it is predictable that rs2278256's association with reduced risk of TNBC is likely to be statistically significant in a larger case number cohort. Importantly, using a promoter reporter assay, our studies showed that the protective alleles of both rs7250266 and rs2278256 had a similar effect on *NBA1* promoter activity, decreasing promoter activity and thus likely to down-regulate NBA1 protein expression levels. Our further analysis indicated that rs7250266 and rs2278256 in *NBA1* were significantly associated with an decreased risk of developing TNBCs, but not with non-TNBC breast cancer susceptibility.

In summary, our study analyzed 37 SNPs in the BRCA1-A complex genes. It is the first study to evaluate genetic susceptibility of the BRCA1-A complex genes to TNBC risk in non-Caucasian female population. Compared to the previous findings from GWAS analysis in European ancestry populations [5, 32], we identified SNP rs7250266 in *NBA1* is associated with reduced TNBC risk, but not non-TNBC risk in Chinese women population. In addition, the haplotypes containing two polymorphisms in *NBA1* (rs7250266 and rs2278256) are related to a lower chance of TNBC developing. Our study also indicates that the protective alleles of the two SNPs lead to reduced promoter activity of *NBA1*. As we know, less than 20% of patients are diagnosed as TNBC among all breast cancer patients [40], it has been difficult to recruit a large cohort of TNBC patients for genetic studies. We have included a significant size of cohort of patients including 414 TNBC cases and 652 non-TNBC cases. Despite genetic variants of other BRCA1-A complex component genes investigated in our study are not associated with TNBC, NBA1 gene appears to be an important contributor to the triple-negative breast cancer risk. Further investigation and validation of these SNPs in larger cohorts may facilitate in predication and prevention of TNBC and in counseling individuals for risk of TNBCs development.

## MATERIALS AND METHODS

### Study participants

All the participants were genetically unrelated Chinese Han women living in Shanghai City and its surrounding areas. All the 414 patients with triple-negative breast cancer included in the step-one study were consecutively diagnosed in Fudan University Shanghai Cancer Center during 2008–2011. 652 patients with non-TNBC breast cancer recruited in the second step study were consecutively diagnosed in Fudan University Shanghai Cancer Center during 2008–2009. All the controls (354 in step-one study and 890 in step-two study) were collected from women who had come to the outpatient department for breast cancer screening. They were determined as cancer-free after comprehensive examination. The brief diagnostic criteria for TNBC are described in Supplementary Methods. This study was approved by IRB-Fudan University Shanghai Cancer Center and all the participants provided informed consent for research participation.

### Polymorphism selection

We selected single nucleotide polymorphism (SNP) in five BRCA1-A complex genes with the following criteria for evaluation: 1) SNPs located in functional regions of the gene, i.e. exons, intron-exon boundaries, promoter region and 3′ UTR, with MAF larger than 0.05. All SNPs located in regions spanning from 2 kb upstream to 0.5 kb downstream of the investigated genes were surveyed in the International HapMap Project database (HapMap Genome Browser release #27, Phase 1, 2 & 3 - merged genotypes & frequencies). 2) tagSNPs based on data provided by Hapmap. The International HapMap Project had genotyped a large number of SNPs in different populations and provided a set of tag SNPs (tagSNPs) which efficiently represent evolutionally linked genetic variants. In this study, tagSNPs were identified using the Tagger Pairwise method with an r^2^ cut off of 0.8 and MAF cutoff of 0.05 in Chinese Han population. SNPs which show significant deviations (*P* < 0.05) from Hardy–Weinberg equilibrium (HWE) among controls or call rate less than 0.95 were excluded in the study. 3) SNPs investigated in previous literatures (hot SNPs).

### DNA preparation and genotyping

Genomic DNA was extracted from peripheral venous blood (3–5 ml) of the individuals in the study group by using Gentra's PureGene DNA Purification kit (Gentra Systems, USA), Genotype data of SNPs were obtained by applying the DNA samples to the SEQUENOM MassARRAY platform (SEQUENOM MassARRAY, San Diego, CA). All samples of cases and controls were seeded randomly with non-template and CEPH controls in each plate for the iPLEX PCR array. The primers used in this study for genotyping were shown in Supplementary Table S1. All polymorphisms were genotyped successfully with genotyping call rate ranging from 95 to 100%. The genotyping experiment was carried out by the Bio-X Life Science Research Institute (Shanghai).

In the step two non-TNBC cases and controls study, PCR-based TaqMan assays (Applied Biosystems, Foster City, USA) of 652 non-TNBC patients and 890 controls were performed on a 7900 HT sequence detector system (Applied Biosystems) according to the manufacture's instructions. Genotyping was automatically attributed using the SDS2.4 software for allelic discrimination. 10% of the samples selected randomly were genotyped again and the reproducibility was 100%.

### Statistical analysis

Results were expressed as percentages for categorical variables. Tests of association were conducted using Pearson's χ^2^ test. Hardy–Weinberg equilibrium (HWE) was tested by χ^2^ tests for each SNP locus. Logistic regression was used to analyze the association between a single locus and breast cancer risk. The odds ratio (OR) and its 95% confidence interval (95% CI) were also shown. The D' and r^2^ statistics were used to assess pairwise LD between SNPs. The Haploview 4.2 program (Broad Institute, Cambridge, MA, USA) was used to test for MAF (minor allele frequency), Hardy–Weinberg equilibrium, linkage disequilibrium (LD) of SNPs in the target genes. SNPs with MAF over 0.01 were included for haplotype evaluation. We used the SNPstat (Catalan Institute of Oncology, Catalonia, Spain) to estimate haplotype frequencies and assess the association between haplotypes and risk of developing triple-negative breast cancer based on the observed genotypes.

### *In silico* analysis of the investigated SNPs

The brief detail of *in silico* prediction is described in Supplementary Methods.

### Luciferase reporter assay for promoter activity

The *NBA1* promoter fragment was constructed by amplifying genomic DNA with rs7250266 (−620C allele) and rs2278256 (−73T allele) based on sequence information of the reference sequence NM_0011033549.1. Briefly, promoter region of *NBA1* (−964 to +308 bp, the transcriptional start site is determined as +1) was amplified and subcloned into a pGL3 basic vector to generate pGL3 −620C/−73T construct. The Quick-ChangeII site-directed mutagenesis kit (Stratagene, CA) was used to generate pGL3 −620C/−73C, pGL3 −620G/−73T and pGL3 −620G/−73C plasmids respectively. The cell lines (MCF-10A, MDA-MB-231 and MDA-MB-468) were obtained from the Shanghai Cell Bank, Type Culture Collection Committee (Chinese Academy of Sciences) and maintained in complete growth medium as recommended by the distributor. The *NBA1* promoter-reporter constructs were transfected into MCF-10A, MDA-MB-231 or MDA-MB-468 cells together with the internal control pRL-CMV. Luciferase activity was measured using the Dual-Glo luciferase kit (Promega, CA). Cells transfected with pGL3-Basic plasmid were used as a mock control. Luciferase activity was measured by a BioTek Microplate reader (BioTek, Inc). Each experiment was conducted in triplicate at least 3 times.

## SUPPLEMENTARY MATERIALS FIGURE AND TABLES


