# Supplementary material for: Genetic evaluation of BRCA1-A complex genes with triple-negative breast cancer susceptibility in Chinese women
Source: Oncotarget. 2016 Feb 1;7(9):9759–72. doi: 10.18632/oncotarget.7112 (PMC4891082; doi:10.18632/oncotarget.7112)
Supplement: Supplementary file 1 [file oncotarget-07-09759-s001.pdf]

Genetic evaluation of BRCA1-A complex genes with triple-negative breast cancer susceptibility in Chinese women

Supplementary Materials

MATERIALS AND METHODS

Study participants

The triple-negative breast cancer diagnoses of patients are determined by the pathologists of the department of Pathology in the Fudan University Shanghai Cancer Center. The H.E staining of tumor tissue slides was performed and used for determining invasive breast carcinoma. A triple-negative breast cancer case was defined as a patient tumor sample negative in Estrogen Receptor (ER), Progesterone Receptor (PR) and HER2 expression in immunohistochemistry (IHC) staining. Patients with HER2 expression status (IHC, score equals to 2+) were subjected to florescence *in situ* hybridization (FISH) test for the HER2 gene amplification. The HER2 over-expression subgroup was defined as FISH positive or IHC staining score equals to 3+. All criteria in this study followed standard procedure which has been described previously. Table S2 presents the baseline characteristics of all the participants in this study. Patients and controls were compared in age, menopause and body mass index. The triple-negative breast cancer cases were more likely to be at younger age at menarche than controls.

In silico analysis of the investigated SNPs

The genetic variants in this study were analyzed using functional prediction software. Two different computational programs, SIFT (http://sift. jcvi.org/) and PolyPhen-2 (http://genetics.bwh.harvard.edu/pph/) were carried out for functional predictions of non-synonymous variants. For SNPs in promoter region, RegulomeDB (http://regulome.stanford.edu/), TFSEARCH (http://www.cbrc.jp/research/db/TFSEARCH.html) and SNPinfo (http://snpinfo.niehs.nih.gov/) were used to find the binding sites of the transcription factors. Three programs, ESE Finder 3.0 (http://rulai.cshl.edu/cgi-bin/tools/ESE3/esefinder.cgi?process = home), RESCUE-ESE (http://genes.mit.edu/burgelab/rescue-ese/) and PESX(http://cubweb.biology.columbia.edu/pesx/) were used to find the ESEs and ESSs in sequence and predict whether the allele change of synonymous SNPs might cause the ESEs or ESS change.

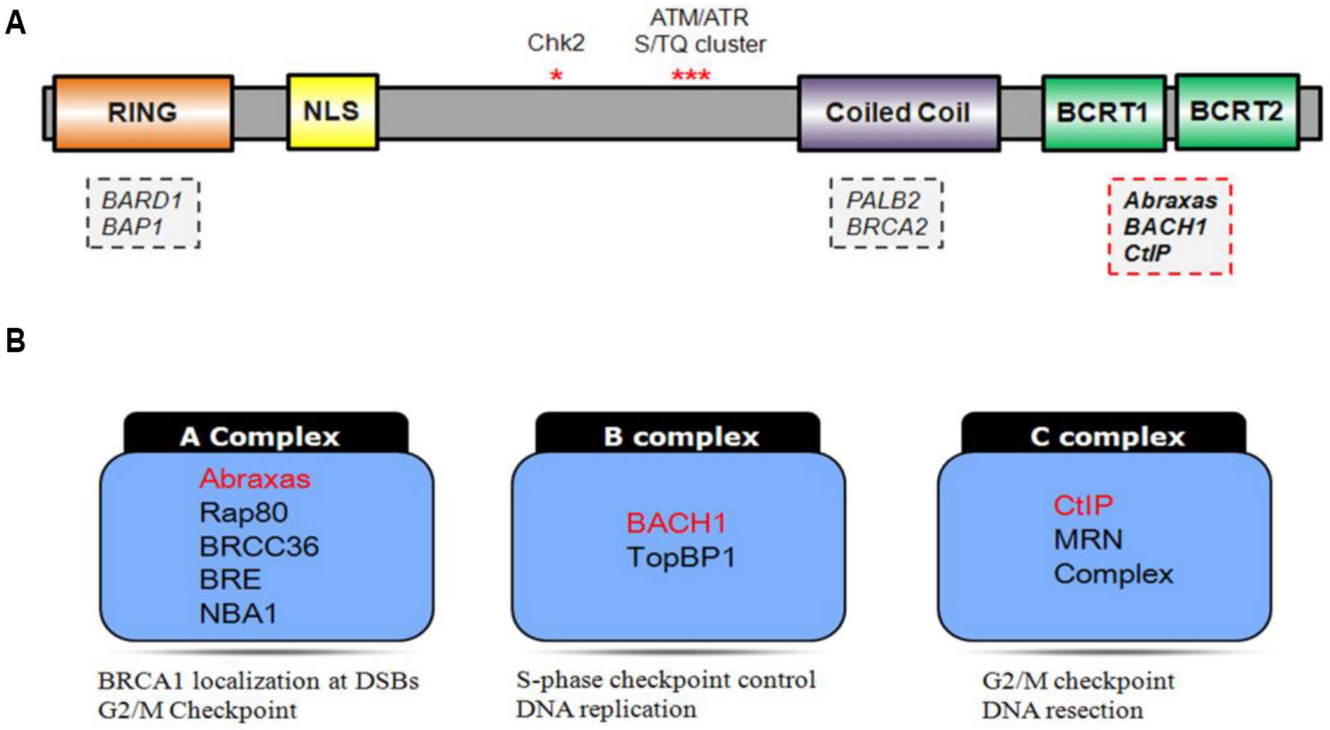

Supplementary Figure: S1 A schematic view of BRCA1 and BRCA1 BRCT domains associated protein complexes. (**A**) BRCA1 contains a RING domain at its N-terminus, a coiled-coil domain and two BRCT domains at the C-terminus. The tandem BRCT domains interact with phospho-SPxF motif-containing proteins ABRAXAS, Bach1 and CtIP. (**B**) A diagram of the BRCA1 BRCT domain associated complexes and their functions. ABRAXAS, BACH1 and CTIP serve as an adaptor protein that binds directly to BRCA1 in the BRCA1 A, B and C complexes. The BRCA1-A complex contains at least five components: ABRAXAS, RAP80, BRE, BRCC36 and NBA1; the BRCA1-B complex contains at least two components: BACH1 and TopBP1; the BRCA1-C complex contains at least CtIP and the MRN complex (Mre11, Rad50 and Nbs1).

Supplementary Table S1: Primers for SNP genotyping

SNP_ID	Primer1	Primer2
rs6721349	ACGTTGGATGAGACCTCTCCTACTCTGATG	ACGTTGGATGTCTGGAATTCTTCTGTCCCC
rs7250266	ACGTTGGATGCCTACCTCTTTTTTCATGGC	ACGTTGGATGTGACTAGCCATGATCGCGTG
rs3745185	ACGTTGGATGAGAGGGACCCATTTGGAGAG	ACGTTGGATGGTATAGGTCATACTGCAGGG
rs13360277	ACGTTGGATGCATCCAGTAACCAGGTATCC	ACGTTGGATGTTGCAGTACATGGCATGTCG
rs10406920	ACGTTGGATGCTGAACAGCCCTTCACTGAC	ACGTTGGATGAGCTGGTACCCTTGGTATCC
rs8100241	ACGTTGGATGGTCCTCTAGCACCAAATTAG	ACGTTGGATGACTCTGACCGCGGTGTTTG
rs58720304	ACGTTGGATGCTCTGAGACCTCTTAAGTTC	ACGTTGGATGCAGAGGGAAGACACTAGTTT
rs12478271	ACGTTGGATGGAGACTTCACTACATCTCTT	ACGTTGGATGGAGGAACTGCACACTTGTTT
rs10403581	ACGTTGGATGTCTCCAAATGGGTCCCTCTG	ACGTTGGATGCATGGCTGAGCAAAGTTCCT
rs10173507	ACGTTGGATGGAGCACATGCCCAACCATTG	ACGTTGGATGTAGGAGACACAGTGAAGCCC
rs6737313	ACGTTGGATGGGCAACAACTCCTCTTTCAG	ACGTTGGATGTTTTCAATGGTTATCTAGG
rs13125836	ACGTTGGATGTGGGCTGCTCATTTTGGATG	ACGTTGGATGCAAGAGATCTCGGTTGTTAG
rs17078630	ACGTTGGATGTGCCCAGACTTCTCTAGACA	ACGTTGGATGCAGTCCAAACGATGGTATGT
rs72931487	ACGTTGGATGACATGCCCAGCCTCAACAAG	ACGTTGGATGGACTGTATGGCCCACAAAAC

rs6710214	ACGTTGGATGACAAGTTAAATGTGGATTG	ACGTTGGATGCATTAATTTTAGGGTGGGAC
rs10209126	ACGTTGGATGCCAGATCACCTAGTCCATAC	ACGTTGGATGTAATGCCTATCATAGTGGCT
rs2278256	ACGTTGGATGAGGCAGCTCTCACTCCAAAC	ACGTTGGATGGAGACAGAAACTGGTTGAGG
rs12642536	ACGTTGGATGCCGAGATCTCTTGAATTGCC	ACGTTGGATGAGCTAGTCCAGCTAGTACAC
rs11891642	ACGTTGGATGTGCAGCCTGGATTTACAATG	ACGTTGGATGCAGGTACCAACAATTGAAG
rs895745	ACGTTGGATGTGTGTAGGGTATTAGGTAGG	ACGTTGGATGCAGTGCTGAACAAAAGAGAC
rs13167812	ACGTTGGATGTAGACAAAAGGATGCCACGG	ACGTTGGATGGAACTGGTAGTTTCCACATC
rs10189899	ACGTTGGATGAACAGGTGAGCAAACCAGAG	ACGTTGGATGCTAGATTTGAGTCTTGCCCC
rs4898413	ACGTTGGATGGGAAACTCCTAGGCAGATAG	ACGTTGGATGAGCCCAACATGAGTTCTAGC
rs353465	ACGTTGGATGTTATGCCATTTGCCTCCCTG	ACGTTGGATGCACAGCATATGCTGAATGTC
rs5945286	ACGTTGGATGAGATGAAGATAAGCACCAGG	ACGTTGGATGTTCACCTTCCCCAGTCAAAC
rs5945300	ACGTTGGATGATGCATCGTTAGGCAACTTC	ACGTTGGATGCATATAGCCTGGGCATGTAG
rs17078658	ACGTTGGATGCACAGCTCTATACAATGCAG	ACGTTGGATGTAAGACAAGTGACTACTCCC
rs17352824	ACGTTGGATGGTGCCTGGGAAAGGTAATGA	ACGTTGGATGGCTGAAAAAATCACTTGTGT
rs6547829	ACGTTGGATGCAGAGAAGGCAACAGATAGG	ACGTTGGATGACTATCTGACCCACCTGTTC
rs8170	ACGTTGGATGAGTTTCGCCATGCAGTTGTG	ACGTTGGATGTGGATACCAAGGGTACCAG
rs12464240	ACGTTGGATGGGTACAACAGCCTTTATGCC	ACGTTGGATGTGAGGCTTCTCAGAGAGAAC
rs12422	ACGTTGGATGGGGACCACTATGGCTTAATG	ACGTTGGATGGAATTTCTAGGGTCCAAAAG
rs3733876	ACGTTGGATGGCTGCTTTGACCAGTAAGAG	ACGTTGGATGGGGTACTTACCAGGACAAAC
rs17709034	ACGTTGGATGAAAAGCCACCTAAACACAAC	ACGTTGGATGTTGTGGCCTTAGTTGTCACC
rs2363956	ACGTTGGATGAGCCATGCAGAGGTGACAAC	ACGTTGGATGGTTTGTCCACAGTTTCAAGG
rs144376330	ACGTTGGATGTAGGGGCACAGGCCAGCGT	ACGTTGGATGTCCTGAAGTGTTGAGGCTCC
rs3734091	ACGTTGGATGAAGATATTCAACAGAGGAG	ACGTTGGATGTGATGAGAGTACTGATGAGG
rs11739147	ACGTTGGATGTTCTTTTTCCTTGCTGTGGG	ACGTTGGATGGTTTTATAAGCTGCTCCTGTC
rs365132	ACGTTGGATGCAAGGTCAAAAGTTTCAGAGG	ACGTTGGATGCACAAAATGAAAATTAATGGG
rs77519137	ACGTTGGATGAAATGTTAACACTCATCAC	ACGTTGGATGATGAGCTATTTGGGGATCAG
rs10420922	ACGTTGGATGTGAGAGCCCCGTATCTTTTG	ACGTTGGATGGAGGGACATTTTAGGGCTTG
rs201627097	ACGTTGGATGGCTTTAGATCGTTTGTCTTG	ACGTTGGATGAGACTTAGATGACAGATGGC

Supplementary Table S2: Clinical characteristic of subjects in step-one cohort (414 TNBCs and 354 controls)

	Patients	Control	P^a value
Age (year)			0.37
≤ 50	256 (61.8%)	230 (65.0%)	
> 50	158 (38.2%)	124 (35.0%)	
Age at Menarche (year)			< 0.01
≤ 13	176 (42.5%)	113 (31.9%)	
> 13	238 (57.5%)	241 (68.1%)	
Menopause			0.84
no	279 (67.4%)	241 (68.1%)	
yes	135 (32.6%)	113 (31.9%)	
Number of Live Birth			0.65
1	364 (87.9%)	315 (89.0%)	
> 1	50 (12.1%)	39 (11.0%)	
BMI			0.62
≤ 24	237 (57.2%)	209 (59.0%)	
> 24	177 (42.8%)	145 (41.0%)	

NOTE: [a] Two-sided χ2 test. $P < 0.05$ was considered statistically significant;
Abbreviations: BMI, body mass index.

Supplementary Table S3: Clinical characteristic of subjects in step-two cohort (652 non-TNBCs and 890 controls)

	Patients	Control	P^a value
Age (year)			0.49
≤ 50	361 (55.4%)	477 (53.6%)	
> 50	291 (44.6%)	413 (46.4%)	
Age at Menarche (year)			< 0.01
≤ 13	261 (40.0%)	182 (20.4%)	
> 13	391 (60.0%)	708 (79.6%)	
Menopause			0.36
no	410 (62.9%)	580 (65.2%)	
yes	242 (37.1%)	310 (34.8%)	
Number of Live Birth			0.09
1	568 (87.1%)	801 (90.0%)	
> 1	84 (12.9%)	89 (10.0%)	
BMI			0.08
≤ 24	372 (57.1%)	497 (55.8%)	
> 24	280 (42.9%)	393 (44.2%)	

NOTE: [a] Two-sided χ2 test. $P < 0.05$ was considered statistically significant;

Abbreviations: BMI, body mass index.

Supplementary Table S4: Associations between each SNPs genotypes and non-TNBCs risk (652 non-TNBCs and 890 controls)

SNP	Genotype	Case	Control	Codominant		Dominant		Recessive	
				OR (95% CI)	*P*	OR (95% CI)	*P*	OR (95% CI)	*P*
rs2278256	TT	288 (44.1%)	401 (45.0%)	reference	0.91	reference	0.74	reference	0.69
	TC	288 (44.1%)	392 (44.0%)	0.98 (0.79–1.21)		0.97 (0.79–1.19)		0.94 (0.68–1.29)	
	CC	76 (11.7%)	99 (11.1%)	0.93 (0.66–1.30)					
rs7250266	CC	434 (66.5%)	600 (67.4%)	reference	0.81	reference	0.71	reference	0.70
	GC	194 (29.8%)	254 (28.5%)	0.94 (0.75–1.18)		0.96 (0.77–1.19)		1.11 (0.66–1.88)	
	GG	24 (3.7%)	36 (4.1%)	1.09 (0.64–1.86)					

Supplementary Table S5: Pathogenicity prediction of selected SNPs

	SNP	Position/ Function	nsSNP		TF Binding Site			miRNA (SNPinfo)		Exonic Splicing Enhancers/ Silencers		
			Poly Phen -2	SIFT	RegulomeDB	TFSEARCH	SNPinfo	miRanda	Sanger	ESE Finder	RESCUE-ESE	PESX
NBA1	rs2278256	5′ near gene			Likely to affect binding and linked to expression of a gene target	change	yes					
	rs7250266	5′ near gene			Likely to affect binding and linked to expression of a gene target	change	yes					
	rs8170	exon/ synonymous								change	ND	ND
	rs144376330	exon/ synonymous								ND	ND	ESS changed
Rap80	rs365132	exon/ synonymous								change	ND	ND
	rs3733876	exon/ missense	Possibly Damaging	Tolerated								
	rs13360277	exon/ missense	Benign	Tolerated								
	rs13167812	exon/ missense	Possibly Damaging	Damaging								
	rs12422	3’UTR						ND	ND			
BRE	rs58720304	3’UTR						change	ND			
Abraxas	rs13125836	exon/ missense	Possibly Damaging	Tolerated								
	rs12642536	exon/ missense	Benign	Tolerated								

ND: no data/ not analyzed

Supplementary Table S6: Summary of literatures

Genes	Reference	Study group	Ethnicity	SNPs (Allele)	Position	Genotype	Haplotype
NBA1	Zheng	patients with breast cancer	African-American	NS		NS	ND
	Solyom	breast/ovarian cancer familial woman	Caucasian	NS		NS	
	Rebbeck	BRCA1 mutation carrier	Mixed (USA)	rs3745185	Intron	HR = 0.82, 0.82	HR = 1.15, 1.22
			(Caucasian/ Hispanic)	rs10406920	Intron	HR = 1.25, 1.37	
				rs8170	Exon/ synonymous	HR = 1.19, 1.24	
	Stevens	patients with TNBC	Caucasian	rs8170	Exon/ synonymous	OR = 1.27	ND
	Antoniou	BRCA1 mutation carrier	Mixed	rs8170	Exon/ synonymous	HR = 1.27, 1.58	ND
		and patients with TNBC		rs3745185	Intron	HR = 0.86, 0.73	
	Present study	Patients with TNBC	Chinese	rs7250266	5′ near gene	OR = 0.77,0.41	OR = 0.73
Brcc36	Rebbeck	BRCA1 mutation carrier	Mixed (USA) (Caucasian/ Hispanic)	NS		NS	NS
	Present study	Patients with TNBC	Chinese	NS		NS	NS
Rap80	Akbari	women with familial breast cancer	Caucasian	c.–24149G > T	5′CpG Island	OR = 2.0[a]	ND
				c.–24001A > G	5′ CpG Island	OR = 2.0[a]	
				c.–8A > G	5′ UTR	OR = 2.0[a]	
				c.*27A > C	3’UTR	OR = 2.0[a]	
	Nikkilä	women with familial breast cancer	Caucasian	c.1304C > T	Exon/ missense	OR = 1.63	ND
	Rebbeck	BRCA1 mutation carrier	Mixed (USA) (Caucasian/ Hispanic)	NS		NS	NS
	Novak	women with familial breast cancer	Caucasian	NS		NS	ND
	Osorio	breast/ovarian cancer familial woman	Caucasian	NS		NS	ND
	Present study	Patients with TNBC	Chinese	NS		NS	NS

Abraxas	Solyom	breast/ovarian cancer familial woman	Caucasian	c.1082G > A	Exon/ missense	OR = 24.3	ND
	Novak	women with familial breast cancer	Caucasian	NS		NS	ND
	Osorio	breast/ovarian cancer familial woman	Caucasian	NS		NS	ND
	Rebbeck	BRCA1 mutation carrier	Mixed (USA) (Caucasian/ Hispanic)	NS		NS	NS
	Present study	Patients with TNBC	Chinese	NS		NS	NS
BRE	Rebbeck	BRCA1 mutation carrier	Mixed (USA)	rs11891642	Intron	HR = 1.26, 1.25	HR = 1.20
			(Caucasian/ Hispanic)				HR = 1.18
	Present study	Patients with TNBC	Chinese	NS		NS	NS

Abbreviations: ND, no data/ not analyzed; NS, no significance

NOTE: [a] The four variants were in complete linkage disequilibrium ($r^2 = 1.0$)
